# Evaluation of the Effects of Paclobutrazol and Cultivation Years on Saponins in *Ophiopogon japonicus* Using UPLC-ELSD

**DOI:** 10.1155/2020/5974130

**Published:** 2020-07-06

**Authors:** Peng Sun, Juhua Tong, Xianen Li

**Affiliations:** Institute of Medicinal Plant Development, Chinese Academy of Medical Sciences and Peking Union Medical College, Beijing 100193, China

## Abstract

Nowadays, there is a growing concern about the quality of herbs used in traditional Chinese medicine. In this study, we evaluated the impacts of paclobutrazol and cultivation period on steroid saponins in *Ophiopogon japonicus*. A rapid method to simultaneously determine three principle steroid saponins (ophiopogonins B, D, and D′) using ultraperformance liquid chromatography combined with an evaporative light-scattering detector was developed. The contents of three saponins in paclobutrazol-treated and nontreated Sichuan *O. japonicus* and those in the 2-year and 3-year Zhejiang *O. japonicus* were analyzed. The results showed that the saponin contents were sharply reduced in paclobutrazol-treated *O. japonicus* as compared to the control, whereas the concentrations of the three targeted saponins in Zhejiang *O. japonicus* varied with the increase in cultivation years, reflecting varied effects on saponins. Our study provided chemical evidences for further quality control and agricultural practices of *O. japonicus*.

## 1. Introduction

In recent decades, there has been a growing popularity of herbal medicines, which can provide polypharmacological effects that orthodox medicines cannot deliver and have been indicated to have distinct therapeutic effects on the treatment of some chronic diseases and metabolic syndromes [1]. It was estimated that 80% of the world's population use herbal medicines as their source of primary care [2]. With the increasing interest in traditional Chinese herbal medicines, the concerns regarding their quality, efficacy, and safety also arise [3, 4]. One critical issue facing traditional Chinese medicines is the different concentrations and proportions of constituents in herbal products, which may lead to great variations in therapeutic results and safety issues. To minimize variation in final herbal products, standardization of procedures should cover the entire field of study from cultivation of herbs to clinical application [5]. And so, the factors that make up the variability in herbs, such as cultivation practice and genotype, must be comprehensively evaluated in the cultivation process.

*Ophiopogon japonicus* (L.f.) Ker Gawl is an evergreen perennial herb in the family Liliaceae that is widely distributed in South China. *O. japonicus* is commonly known as “maidong” in Chinese, and its tuberous roots have long been used to treat coughs, sore throats, thirstiness, constipation, and insomnia in traditional Chinese medicine [6, 7]. *O. japonicus* is largely produced in Zhejiang and Sichuan Province in China, where *O. japonicus* is usually called Zhe maidong (ZMD) and Chuan maidong (CMD), respectively. Though ZMD and CMD both originated from *O. japonicus*, they display different appearances and qualities. Both ZMD and CMD have a yellowish spindle-like shape, but the former is usually thinner and darker than the latter. To distinguish between ZMD and CMD and evaluate their qualities, several analytic and pharmacological studies had been carried out [8–12]. These studies exhibited that CMD and ZMD possessed different chemical constituents and pharmacological activities that would play pivotal roles in clinical efficacy of *O. japonicus* and its derived preparations, implying that the geographical origin of *O. japonicus* is a key factor for the differences in CMD and ZMD.

Besides geographical location, other factors including cultivation practice and genotype also significantly affect the quality of herbs, as has been observed in several herbal species [13, 14]. This is also the case in *O. japonicus*. In practice, ZMD demands a long growing period to be harvested, which is usually harvested in the third year after planting, while CMD is often harvested in the second year as which grows faster in Sichuan. Furthermore, CMD is usually treated with plant regulator paclobutrazol during its growth cycle to achieve high yielding and good commercial grade. Paclobutrazol is a kind of plant regulator that can reduce plant height, improve stem diameter and leaf number, alter root architecture, and directly contribute to yield increase [15]. Currently, the effects of agronomic factors on *O. japonicus* have attracted researchers' interests. Li et al. and Lin et al.'s studies showed that fertilizers and regulators influenced the agronomic character, yield, and appearance quality of *O. japonicus* [16, 17]. Among the agronomic factors, paclobutrazol plays a prominent role in regulation of the growth and metabolism of *O. japonicus*. Li et al. and Zhan et al. reported that paclobutrazol not only affected the growth characteristics of aerial parts but also significantly improved the tuberous root number, size, and dry matter [18, 19]. Furthermore, two recent studies have demonstrated that application of paclobutrazol influenced the total contents of saponins, flavonoids, and polysaccharides (i.e., the main active compositions in *O. japonicus*) [20, 21]. However, the study on the impact of agricultural practices on *O. japonicus* is still limited. It will be interesting and important to differentiate the detailed differences of constituents caused by the influencing factors.

Steroid saponins are the mostly studied active ingredients in *O. japonicus*, and this group of compounds exhibits broad biological activities such as cardiovascular protection, anti-inflammation, anticancer, antioxidation, immunomodulation, and antitussive [22]. According to the Chinese Pharmacopoeia (2015 Edition), the total content of saponins (calculated as ruscogenin) is used as a quality control index of *O. japonicus* and the UV spectrometric method is stipulated for the quantitative determination of the total content of saponins [23]. As many substances in plants have UV-like absorption contours, UV spectrophotometry cannot serve as an ideal approach for the identification of steroid saponins, and there has been increased interest in establishing more precise and accurate methods for the quantification of saponins in *O. japonicus* in recent years. Presently, high-performance liquid chromatography (HPLC) remains the most common technique for the quality assessment of *O. japonicus*. Several HPLC-based methods to determine steroid saponins have been reported [8, 24, 25]. However, the separation of steroid saponins using HPLC (concerning resolution and analysis time) was not ideal due to their highly similar structures and high molecular weights, which cannot well meet the fast and accurate requirements for the quality assessment of *O. japonicus* from different sources. Hence, it is crucial to establish a rapid and reliable analytical method to analyze the steroid saponins in *O. japonicus*. Ultraperformance liquid chromatography (UPLC) has now become a powerful analytical technique that enables to rapidly separate targeted compounds with higher sensitivity and selectivity than HPLC. More recently, UPLC tandem with time-of-fight mass spectrometry has been used to identify isomers of saponins from *O. japonicus* [26]. In the present study, a rapid ultraperformance liquid chromatography with evaporative light-scattering detection (UPLC-ELSD) method for simultaneous determination of ophiopogonins B, D, and D′ was established; this method was validated and then successfully used for evaluation of the effects of paclobutrazol and cultivation years on saponin contents. The results would broaden our knowledge on the phytochemical variation in cultivated *O. japonicus* under different cultivation practices.

## 2. Materials and Methods

### 2.1. Plant Materials

CMD and ZMD were, respectively, collected from Santai county, Sichuan Province, and Cixi city, Zhejiang Province of China. CMDs were divided into two groups: paclobutrazol-treated group and control group. In the first year, the paclobutrazol group was sprayed with paclobutrazol in October. Two groups of CMDs were harvested in the second year. ZMDs were divided into two types: the yellow-leafed type (YL) and green-leafed type (GL). YL *O. japonicus* is a variant of GL *O. japonicus* (the most common *O. japonicus*). ZMDs were separately harvested in the second and third years. All samples were washed, deenzymed at 105°C for 15 min, and then dried at 60°C. All plant materials were authenticated by Prof. Xianen Li (Institute of Medicinal Plant Development, Chinese Academy of Medical Sciences). The information about all the samples mentioned above is summarized in Table 1.

### 2.2. Chemicals and Regents

The reference compounds ophiopogonin B and ophiopogonin D′ were purchased form Sichuan Weikeqi Biotech Co., Ltd. (China), while ophiopogonin D was from Shanghai Yilin Biotech Co., Ltd. (China). The purity of standards was higher than 98%. HPLC grade acetonitrile was from Fisher Scientific (USA). Water used for all analyses was from Wahaha Co. (China). The methanol and butanol used for plant extraction were of AR grade from Beijing Chemical Corporation (Beijing, China). All other chemicals were of reagent grade.

### 2.3. Standard Preparation

The reference compounds ophiopogonins B, D, and D′ were accurately weighed and dissolved in 1 mL methanol to produce a mixed standard stock solution with the concentrations of 0.2013 mg/mL, 0.2813 mg/mL, and 0.2125 mg/mL, respectively. 2, 4, 8, 12, 16, and 20 *μ*L of the standard mixture were separately taken and diluted in methanol for UPLC-ELSD analysis to make calibration curves.

### 2.4. Sample Preparation

Two grams of *O. japonicus* powder was extracted under reflux with 100 mL methanol for 1 hour and filtered. The extract was evaporated to dryness in a rotary evaporator at 65°C under reduced pressure and redissolved in 50 mL of water. The obtained solution was subsequently extracted four times with water-saturated butanol (25 mL, 25 mL, 20 mL, and 20 mL each). The pooled butanol portions were washed twice with 5 mL 10% ammonium hydroxide solution. The butanol fraction was then evaporated to dryness at 65°C under the reduced pressure and dissolved in 5 mL methanol for the below UPLC-ELSD analysis.

### 2.5. UPLC Conditions

A Waters Acquity UPLC system (Waters, USA, including Waters 2707 autosampler and Empower 2 chromatography workstation) coupled with an evaporative light-scattering detector was used for sample analysis. The separation of samples was performed on an Acquity UPLC BEH C18 column (2.1 mm × 100 mm, 1.7 *μ*m, Waters, USA) maintained at 30°C. The injection volume was 5 *μ*L. The mobile phase consisted of (A) acetonitrile and (B) water. The UPLC elution conditions were optimized as follows: 45% A 0-1 min, 45%–49% A 1–3 min, 49% A 3–5 min, 49%–51% A 5–8 min, 51% A 8-9 min, and 51%–55% A 9-10 min, and then, the column was returned to 45% A for the next injection. The flow rate was set at 0.20 mL/min. The ELSD settings were as follows: drift tube temperature, 90°C; nitrogen flow rate, 2.07 mL/min; and heating power level of the sprayer, 60%. Three replicates of each sample were measured.

## 3. Results and Discussion

### 3.1. Method Development

**Figure 1:**
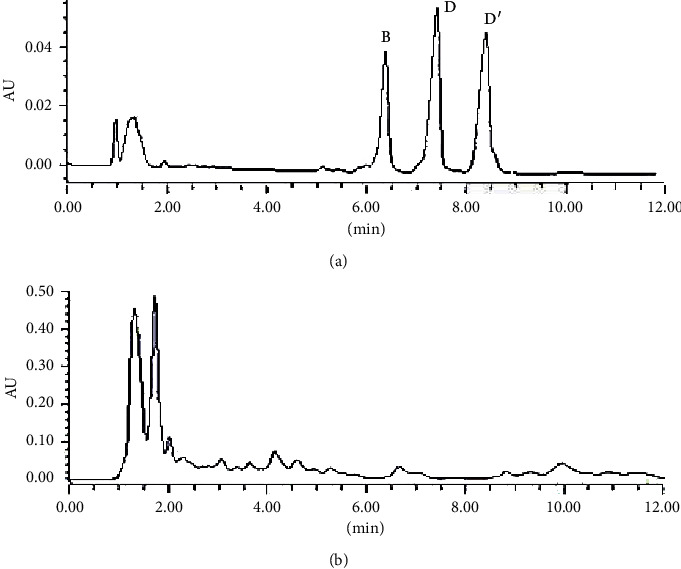
UPLC-UV chromatographs of standard mixture (a) and maidong extract (b) at 208 nm.

**Figure 2:**
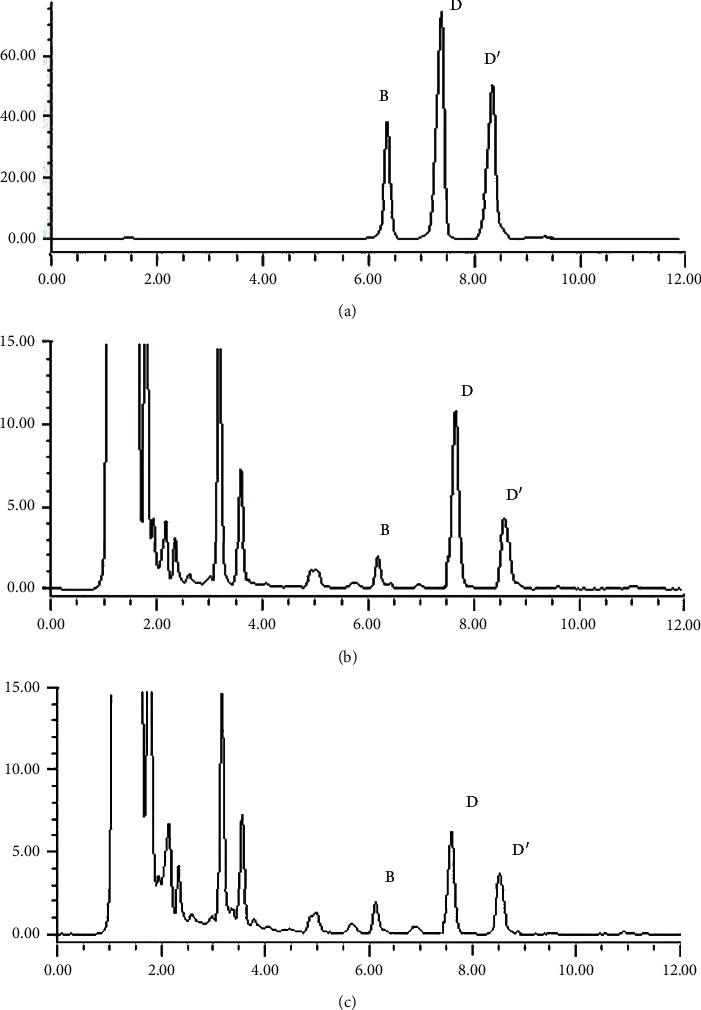
UPLC-ELSD chromatographs of standard mixture (a), CMD extract (b), and ZMD extract (c).

Like almost all other saponins, ophiopogonins lack a chromophore. Although saponins absorb UV light at a wavelength below 210 nm, due to their low concentrations, they were undetectable in *O. japonicus* samples under the condition of UV detection at 208 nm (Figure 1). In literature, quantification analysis of saponins in *O. japonicus* was generally performed by means of HPLC-ELSD detection based mainly on C18 columns under gradient elution [8, 24, 25]. However, because of the high similarity of their chemical structures, performing a baseline separation of the ophiopogonins using the HPLC method was found to be difficult. Even under conditions of long elution time and high flow rate, the peaks were not well resolved, making the HPLC method time-consuming and imprecise. In the present study, the developed UPLC method ensured sufficient chromatographic separation and accurate and precise quantification of ophiopogonins B, D, and D′ (Figure 2). Moreover, the use of ELSD detector, a common detector in most labs, makes the developed method suitable for routine quality control of *O. japonicus*.

At the stage of the method development, the extraction methods: heat reflux extraction, maceration, and ultrasound-assisted extraction, were applied for the extraction of saponins from *O. japonicus*. A maximum saponin yield of 0.274 mg/2 g DW could be obtained in 2 h by heat reflux extraction (2 g of sample: 100 mL of methanol, 70°C) compared to 24 h for maceration (0.185 mg/2 g DW; 2 g of sample: 100 mL of methanol, 25°C) and 2 h ultrasound-assisted extraction (0.257 mg/100 mg DW; 2 g of sample: 100 mL of methanol, 25°C). Therefore, the heat reflux extraction technique, which achieved the best yields of saponins without consuming too much time, is suitable compared with maceration and ultrasound-assisted extraction methods. In heat reflux extraction using water, ethanol, and methanol solvents, methanol displayed better extraction efficiency (0.131 mg/g) than ethanol and water, the yield obtained from water extraction was especially low (0.056 mg/g), and thus, methanol was selected as extraction solvent. Moreover, there was no correlation between the extraction time (1 h, 2 h, 3 h, and 4 h) and the extraction yield of saponins; 1 h was found to be adequate for the analysis. And so, heat reflux extraction using 100 ml methanol for 1 h was adopted in this study for the subsequent analysis.

### 3.2. Methods Validation

The current UPLC-ELSD method was validated for its linearity, precision, repeatability, and stability effects. The linear regression equations obtained for three analytes were *y* = 1.6489*x* + 5.1185 (ophiopogonin B, *R*^2^ = 0.9994), *y* = 1.4937*x* + 5.3108 (ophiopogonin D, *R*^2^ = 0.9992), and *y* *=* 1.5438*x* + 5.2992 (ophiopogonin D′, *R*^2^ = 0.9993), respectively, suggesting a good relationship between concentrations and peak areas of the analytes within the test ranges. A sample was assayed successively for 6 times to evaluate the instrumental precision. The RSDs of peak areas were less than 3% (2.61%, 2.42%, and 1.80%, respectively), indicating that the analysis instrument was in good precision condition. Six samples from the same batch were prepared and analyzed by the developed method for repeatability assessment; the RSDs for ophiopogonin B, ophiopogonin D, and ophiopogonin D′ were 1.76%, 2.04%, and 2.57%, respectively, indicating that the developed method was with acceptable repeatability. For the stability test, the RSDs of peak areas for ophiopogonins B, D, and D′ detected by the method were 2.36%, 2.93%, and 2.05% within 24 h, respectively, reflecting that this method was stable. The established method also had acceptable accuracy with spike recovery, which were 99.63%, 101.67%, and 100.45% for ophiopogonins B, D, and D′, respectively. Finally, this method was successfully employed for quantitative determination of ophiopogonins B, D, and D′ in CMD and ZMD samples.

### 3.3. Effect of Paclobutrazol on Saponin Contents

**Figure 3:**
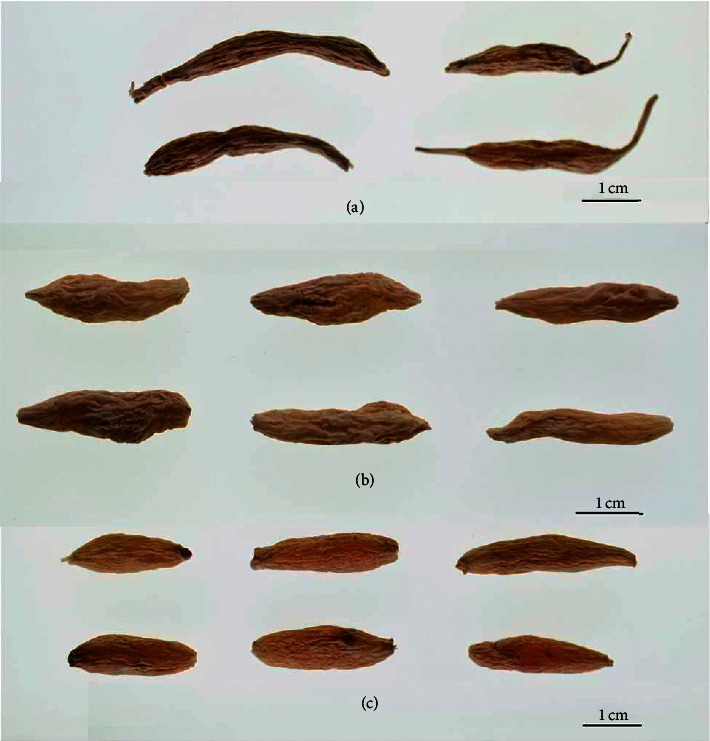
Samples of ZMD (a), CMD (b), and paclobutrazol-treated CMD (c).

**Figure 4:**
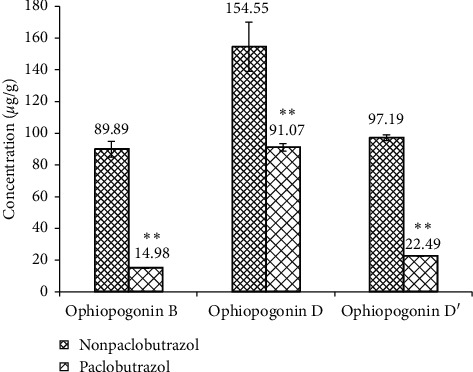
The concentrations of ophiopogonin B, ophiopogonin D, and ophiopogonin D′ in paclobutrazol-treated CMD and nontreated samples. ^*∗∗*^*p* ≤ 0.01.

In the decade, CMD is becoming the dominant species in maidong market due to its high yield and appearance quality. During growing season, paclobutrazol is often applied on CMD to achieve economic efficiency. Paclobutrazol-treated CMD samples have a glossy appearance and are plumper compared with nonpaclobutrazol-treated CMD samples, as shown in Figure 3. However, the influence of paclobutrazol on the effective constituents in *O. japonicus* needs to be comprehensively evaluated for medicinal purpose. In this study, we assessed the effect of paclobutrazol on the major saponins in *O. japonicus* using UPLC-ELSD. The concentrations of ophiopogonins B, D, and D′ in blank and paclobutrazol-treated CMD samples were determined and shown in Figure 4. The results showed that the contents of three saponins in CMD were dramatically reduced by using paclobutrazol, especially for those of ophiopogonins B and D′, which, respectively, decreased by 83.33% and 76.86% as compared to the controls. These findings suggest that paclobutrazol plays a significantly negative role in contributing to the accumulation of three targeted saponins in *O. japonicus*. It is also of no surprise that paclobutrazol-treated CMDs had much lower total saponin content than controls, and the average concentrations of treatment group and the control group were 128.54 *μ*g/g and 341.63 *μ*g/g, respectively. This is consistent with the previous studies reported by Lin et al. and Zhang et al. in which the total saponin contents in *O. japonicus* were reduced by the application of paclobutrazol [20, 21].

### 3.4. Influence of Growing Period on the Saponin Contents of ZMDs

The varied amounts of three saponins in the 2-year and 3-year ZMDs are shown in Table 2. The contents of ophiopogonin D in *O. japonicus* sharply increased with the cultivation years, in which GL *O. japonicus* increased from 18.64 *μ*g/g in the 2nd year to 41.51 *μ*g/g in the 3rd year and YL *O. japonicus* was from 2.45 *μ*g/g in the 2nd year to 11.29 *μ*g/g in the 3rd year. It seemed that the increase in growth period had a very weak effect on the accumulation of ophiopogonin D′, which slightly increased in both types of *O. japonicus* within the 3rd year. As for ophiopogonin B, the content variation associated with growth was significantly different in the two genotypes. The mean amount of ophiopogonin B in GL *O. japonicus* declined from 52.64 *μ*g/g in the 2nd year to 36.79 *μ*g/g in the 3rd year, while that in YL *O. japonicus* slightly increased from 16.33 *μ*g/g in the 2nd year to 17.56 *μ*g/g in the 3rd year. Besides, it is noteworthy that ophiopogonins B, D, and D′ were lower in YL *O. japonicus* compared with GL *O. japonicus*, suggesting the impact of genotype on saponins.

Our results revealed that cultivation period and genotype played a definite role in influencing the content of saponins in *O. japonicus*, providing important information on the standardization of cultivating *O. japonicus*. However, the interaction effects of cultivation period, genotype, paclobutrazol, and other environmental factors such as geographical location on the accumulation of saponins in ZMD and CMD are still not clear, and this will be one of the important research aspects that needs more detail investigation in future.

## 4. Conclusions

A reliable UPLC-ELSD method was established to simultaneously determine ophiopogonins B, D, and D′ in *Ophiopogon japonicus*. Our results demonstrated that the paclobutrazol treatment sharply decreased the content of ophiopogonins B, D, and D′ in CMDs. The content of ophiopogonin D in the 3-year ZMDs was significantly higher than that in the 2-year ZMDs for both genotypes. As for ophiopogonins B and D′, the two genotype ZMDs showed variable changes in response to growth. Our results also provided the information on the quality control of *O. japonicus* by using standard cultivation practices and selecting desirable varieties containing a higher amount of desired saponins in future.

## Figures and Tables

**Table 1:** Summary of the samples used in the present study.

Origin	Genotype	Developmental stage	Paclobutrazol treatment	No. of samples
CMD	GL	2nd year	Yes	5
			No	10

ZMD	GL	2nd year	No	5
		3rd year	No	10
	YL	2nd year	No	3
		3rd year	No	3

**Table 2:** The mean contents of saponins in the 2-year and 3-year ZMD samples (*μ*g/g).

	GL		YL	
	2Y	3Y	2Y	3Y
Ophiopogonin B	52.64 ± 5.45	36.79 ± 0.22	16.33 ± 0.84	17.56 ± 0.32
Ophiopogonin D	18.64 ± 0.60	41.51 ± 2.12^*∗∗*^	2.45 ± 0.72	11.29 ± 0.38^*∗∗*^
Ophiopogonin D′	62.02 ± 10.34	65.90 ± 2.64	14.32 ± 0.29	15.63 ± 0.35
Total	133.33	144.20	33.10	44.48

^*∗∗*^Significant difference in contents between 2-year and 3-year maidong (*p* ≤ 0.01). GL: green leaf maidong; YL: yellow leaf maidong; 2Y: 2-year maidong; 3Y: 3-year maidong.

## Data Availability

The data used to support the findings of this study are available from the corresponding author upon request.
